# Development of a Nutrition Questionnaire for Dental Caries Risk Factors

**DOI:** 10.3390/ijerph17051793

**Published:** 2020-03-10

**Authors:** Sara A. Patenaude, Petros Papagerakis, Jessica R.L. Lieffers

**Affiliations:** 1 College of Pharmacy and Nutrition, University of Saskatchewan, Saskatoon, SK S7N 5E5, Canada; s.patenaude@usask.ca; 2 College of Dentistry, University of Saskatchewan, Saskatoon, SK S7N 5E4, Canada; petros.papagerakis@usask.ca

**Keywords:** diet, dental caries, surveys and questionnaires, nutrition assessment, cross-sectional studies, dental care

## Abstract

Background: Few existing tools quickly identify dietary behaviours related to dental caries. The objectives of this study were to (i) create a patient-generated questionnaire identifying these dietary behaviours, (ii) capture information on these dietary behaviours in two specific populations via questionnaire pilot testing and (iii) determine questionnaire test-retest reliability. Methods: After development, the questionnaire was reviewed by an expert panel. Cognitive interviewing was conducted, followed by pilot testing in a general university campus population (*n* = 80) and a university dental clinic (*n* = 10). Retesting was done with the general campus group (*n* = 53). Results: Most participants reported never receiving dietary advice from professionals regarding caries. Sugary foods were most often consumed as snacks in the evening or afternoon, then breakfast. In total, 41.3% of campus participants consumed high risk items at least a few times per week or more often. Weekly or more frequent consumption of “other” sugary drinks (e.g., iced tea) was common. In total, 77.6% of questionnaire items had a kappa value representing moderate agreement or greater. Conclusions: Dietary behaviours related to caries were common in this pilot study. Given the high prevalence of caries and low occurrence of prior dietary advice for the same, increased preventive efforts may be warranted.

## 1. Introduction

Dental caries is one of the most common preventable chronic diseases in the world [1]. Untreated dental caries (also called dental cavities or tooth decay) affect 2.4 billion people worldwide [2]. The burden of dental caries is also substantial in Canada. Data from the 2007–2009 Canadian Health Measures Survey found that 96% of adults in Canada have a history of dental caries [3]. Fluoride application can be used to treat early stages of disease; however, many cases require more invasive and expensive restorative treatments (fillings and dental crowns) with the most severe cases requiring extraction. Cases where decay has spread to the pulp of the tooth may also require pulpectomy or root canal therapy. In addition, the treatment of severe cases of dental caries in young children sometimes requires general anesthesia; in fact, this was the most common day surgery procedure in children aged 12–59 months in Canada from 2010–2014 [4].

The role of the diet in the development of dental caries is not simply one contributor to a multi-factorial disease process [5]; rather, dietary patterns and choices either provide or do not provide fermentable carbohydrates (including sugars), which are the major essential precursor to caries formation [6]. The caries process occurs dynamically as bacteria (e.g., *Streptococcus mutans*) in the mouth utilize free sugars—chiefly sucrose—as an energy substrate. When fermentable carbohydrates are used for energy, pH-lowering acid is produced as a metabolic byproduct. When the critical pH of approximately 5.5 is reached, the tooth enamel and exposed dentin become demineralized. Over time, if this process repeats to the extent and frequency that enamel is not able to sufficiently re-mineralize between acid exposures, caries are formed [7,8].

While preventive strategies such as fluoride (in water, toothpaste, and via in-office procedures) have been shown to effectively reduce the speed at which carious lesions develop, they do not address the principal underlying cause of the disease process, which is the intake of dietary sugars [9]—without this, caries would not occur. Multiple clinical and population-based studies worldwide have found a direct correlation of caries burden with the free sugar content of the diet [5]. While consequences for children can be particularly devastating, the progressive nature of dental caries dictates an urgent need for dietary intervention strategies and tools directed at individuals of all ages [5,9].

Despite the extensive body of knowledge on the process, prevalence, and consequences of dental caries, few tools have been created for dietitians, dental professionals, and other healthcare educators to quickly and efficiently assess dietary habits to identify nutritional risk for dental caries [10,11,12,13]. Although a large body of long-term evidence to support the effectiveness of nutrition counselling on caries reduction is lacking [14], it has been demonstrated that nutrition counselling may achieve significant reductions in caries rates and reduce high risk dietary behaviours [13,15]. Commonly used diet reporting techniques include food diaries, food frequency questionnaires and food recalls [14], which require nutritional knowledge, training, and clinician time to assess. Because of these factors, it is unsurprising that reported barriers to dietary assessment and counselling in the dental setting include insufficient compensation, lack of personal knowledge needed for diet analysis and counselling, and negative attitudes towards the usefulness of such tools [16,17].

Client-generated assessment tools may address clinician barriers by requiring minimal time and training to use. To our knowledge, no such questionnaire focusing exclusively on dietary behaviours that are both protective and harmful regarding dental caries for individuals of all ages currently exists. The purpose of this study was (i) to create a questionnaire that identifies dietary behaviours related to dental caries and can be expediently completed by the client, (ii) to conduct a pilot test of the questionnaire to capture information on dietary behaviours related to dental caries in two different populations and (iii) to assess test-retest reliability of the questionnaire.

## 2. Materials and Methods

The study was approved by the University of Saskatchewan Behavioural Research Ethics Board (BEH: #99). All participants provided informed consent.

### 2.1. Questionnaire Development and Expert Review

A questionnaire was drafted to reflect current evidence on dietary behaviours influencing risk of dental caries in April 2018–May 2018. The goal was to create a tool that captures data on risk factors, protective factors and overall quality of the diet within the past 3 months that can be expediently completed by the client. First, questions were included to assess dietary behaviours that have been positively associated with increased development of dental caries. The timing, frequency of intake and total intake of foods containing fermentable carbohydrates and the direct addition of dietary free sugars have been associated with proportional increase in risk of caries. Some forms of fermentable carbohydrate (e.g., sticky textures, highly processed starches) have also been implicated in increased risk of caries [7,8,18]. Second, questions were included to capture data on dietary behaviours that may provide protection against caries, particularly when consumed with or after fermentable carbohydrates, including dairy products (e.g., milk, cheese and yogurt) [7,8]. Third, questions regarding general dietary patterns were developed to assess overall frequency of food and beverage intake, as risk of dental caries has been shown to increase proportionally to frequency of dietary intake in general. Vegetable and fruit intake was assessed as an indicator of overall diet quality [7,8,18].

After the development of a draft questionnaire, an expert panel of Canadian dental (*n* = 2) and nutrition (*n* = 5) professionals from academic, clinical and public health backgrounds reviewed the document in May 2018. Reviewers were invited to provide comments on specific questions as well as the overall questionnaire, and to score each item for content validity and clarity, with response options given as 1 = “not”, 2 = “somewhat”, 3 = “rather” or 4 = “highly” relevant or clear. Responses were summed and averaged to determine a mean score for the relevancy and clarity of each question, and ranged from 3.29 to 4.00 and 2.33 to 4.00, respectively. One question with a low mean score was removed and revisions were made.

### 2.2. Cognitive Interviews

Following the expert review, cognitive interviews [19] were conducted with adults (*n* = 4) from the University of Saskatchewan campus community in June 2018. Participants were female undergraduate students or staff members with a university degree. These participants were provided with a thank you gift card ($10 CAD).

During the interviews which ranged in duration from 15–30 min each, participants were instructed to complete the questionnaire by “thinking out loud” as they read and answered each question. Commentary was encouraged around interpretation of wording, calculations and arrival at answers, and available response options. Comments were recorded and later analyzed to identify common themes.

These findings revealed general confusion regarding cariogenicity of sugars perceived to be naturally-occurring vs. chemically processed. In addition, comments were used to revise the questionnaire to have more precise wording around dietary sugars, portion sizes and specific food or beverage items.

### 2.3. Pilot Testing

Next, the questionnaire was pilot tested concurrently in two groups at the University of Saskatchewan from June 2018–July 2018: the general campus community (campus group—online survey mounted on SurveyMonkey (SurveyMonkey Inc, San Mateo, CA, USA)), and patients attending the University of Saskatchewan Dental Clinic (clinic group—paper-based survey). Nutrition and dental students, faculty and professionals were not eligible to participate. Participants were recruited from the campus community through an advertisement on the university’s online bulletin system. Patients attending the university’s dental clinic were invited to participate by clinic reception staff. In addition to completing the nutrition questionnaire, participants were also invited to provide demographic, dental health and oral hygiene information. During the pilot test, participants were also invited to provide constructive feedback on the nutrition questionnaire. A formal sample size calculation was not conducted for the pilot test as Thabane et al. [20] have suggested that sample size calculations for pilot studies are not always necessary.

### 2.4. Test-Retest Reliability

All participants from the campus group were invited to participate in test-retest reliability testing of the nutrition questionnaire. A sample size of >50 was desired for the test-retest reliability study [21]. If interested, participants were sent a link by email to complete the nutrition questionnaire a second time approximately one week later. A $10 CAD gift card was offered as compensation to those who did. A copy of the final version of the nutrition questionnaire is available as supplementary information in Figure S1.

### 2.5. Data Analysis

All data analyses were conducted using SPSS v25 (IBM Corp, Armonk, NY, USA). Descriptive statistics were performed for the first questionnaire completion for all participants from both groups.

To measure test-retest reliability, both absolute (percentage) agreement as well as kappa (Cohen’s kappa for nominal variables; weighted kappa with linear weights for ordinal variables) were determined. The extension STATS_WEIGHTED_KAPPA was used to determine weighted kappas in SPSS. We considered kappas of <0.20 to represent slight agreement, 0.21–0.40 fair agreement, 0.41–0.60 moderate agreement, 0.61–0.80 substantial agreement, and 0.81 or greater to represent almost perfect agreement [22].

## 3. Results

In the campus group, *n* = 90 consented to participate, and *n* = 80 completed most or all of the nutrition questionnaire. In the dental clinic group, *n* = 10 participants completed most or all of the nutrition questionnaire. Participant demographics are provided in Table 1. The sample was mainly female (campus: 75.0%; clinic: 70.0%) and most campus group participants were students (undergraduate: 45.0%; graduate: 33.8%). In both groups, most participants identified as Caucasian (campus: 72.5%; clinic: 75.0%). In addition, about 1/5 of participants were newcomers to Canada within the previous five years (campus: 16.3%; clinic: 22.2%). Most participants also self-reported a history of dental caries (campus: 85.9%; clinic: 77.8%), with almost half self-reporting having had caries diagnosed by a dental professional within the past two years (campus: 41.8%; clinic: 42.9%).

Many participants indicated that they had never previously received nutrition information or advice from a health professional regarding dental caries (campus: 62.8%; clinic: 44.4%). Of those participants who had received advice from a health professional, most had received information from a dental professional (campus: 83.3%, clinic: 100.0%), followed by a registered dietitian (campus: 11.1%, clinic: 40.0%), doctor (campus: 2.8%, clinic: 20.0%), or a nurse/nurse practitioner (campus: 2.8%, clinic: 20.0%).

Participant self-reported behaviours regarding intake of sugary foods are presented in Table 2. Evening snacks, afternoon snacks, and breakfast were the most common times for sugary food consumption among participants. In the campus group, 51.3%, 40.0%, and 33.8% of participants usually consumed sugary foods at evening snacks, afternoon snacks, and breakfast, respectively. In the clinic group, 70.0%, 30.0%, and 20.0% of participants usually consumed sugary foods at afternoon snacks, evening snacks, and breakfast, respectively. In the clinic group, most people (60.0% of participants) reported adding sugar to food and drinks, whereas in the campus group, most people indicated that they did not (57.0% of participants). Among those who added sugar to their food and drinks, the reported mean amount added was 2.0 ± 1.3 teaspoons/day in the campus group and 2.1 ± 1.7 teaspoons/day in the clinic group. Considering high risk items (e.g., dried fruit/fruit snacks, sugary candies, mints or lozenges), 41.3% of the campus group reported consuming these items a few times per week or more often versus 40.0% of the clinic group.

Foods and beverages were commonly reported to be consumed as snacks by participants; these items were examined separately due to differences in clearance times of liquids and solids in the oral cavity. In the campus group, for morning snacks, food was more frequently reported to be usually consumed compared to beverages (40.0% of participants reported usually consuming food vs. 8.8% of participants reported usually consuming beverages). However, in the clinic group, 10.0% of participants reported consuming food and 10.0% of participants reported consuming beverages at morning snacks. At evening snacks, more participants in both groups usually consumed food compared to beverages (campus: 53.8% food vs. 36.3% beverages; clinic: 50.0% food vs. 20.0% beverages). In the clinic group, for afternoon snacks, 40.0% of participants reported usually consuming beverages and 30.0% reported usually consuming food. However, in the campus group, 52.5% reported usually consuming food, and 23.8% reported usually consuming beverages for afternoon snacks. On average, the campus group reported consuming 2.7 servings of vegetables and 2.1 servings of fruit daily. The clinic group reported consuming 2.3 and 2.1 servings of each, respectively.

Behaviours regarding intake of beverages and foods that may increase or decrease caries risk are reported in Table 3. For sugary non-dairy beverages consumed, “other” sugary drinks (e.g., iced tea, sports or energy drinks, sweetened iced coffee, iced cappuccinos, etc.) were commonly consumed in both groups (campus: 53.8% of participants consumed these beverages a few times per week or more often; clinic: 50.0% consumed these beverages a few times per week or more often). Juice was more commonly consumed compared to pop in both groups (juice - campus: 36.7% consumed juice a few times per week or more often; clinic: 50.0% consumed juice a few times per week or more often; pop – campus: 20.3% consumed pop a few times per week or more often; clinic: 30.0% consumed pop a few times per week or more often). Just 7.9% of the campus and 20.0% of the clinic participants consumed flavoured cow’s milk a few times per week or more often, while 44.7% and 66.7%, respectively, consumed sugar-sweetened yogurt a few times per week or more often. Soy milk was consumed infrequently; it was not consumed in the clinic group, and only 6.5% of campus participants reported consumption a few times per week. No participants in the clinic group reported consuming “other” non-dairy milk beverages, however, 33.3% of the campus group participants reported consuming these beverages a few times per week or more often. Just 23.4% of campus participants reported consuming plain cow’s milk at least once per day or more frequently, compared to 50.0% of the clinic group. For cheese, 79.5% of campus and 90.0% of clinic participants reported consumption a few times per week or more often; for unsweetened yogurt, 38.2% of campus participants and 60.0% of clinic participants reported consumption a few times per week or more often.

In total, 60 campus group participants began the nutrition questionnaire a second time, approximately one week after their first completion; of those, *n* = 53 completed the questionnaire and had their first and second responses successfully matched. Detailed test-retest reliability results are available in Table 4. Overall, absolute (%) values ranged from 50.9% to 96.2%, Weighted Kappa values ranged from 0.503 to 0.834 and Cohen’s Kappa values ranged from −0.026 to 0.767. In total, 77.6% of questionnaire items had a value of 0.41 (moderate agreement) or greater. By question type, 69.4% of Cohen’s kappa scores (used for categorical variables), and 100% of weighted kappa scores (used for ordered responses) achieved moderate agreement or greater.

## 4. Discussion

To our knowledge, this is among the first studies to describe the development, pilot testing and test-retest reliability testing of a patient-generated questionnaire capturing information exclusively on a variety of dietary behaviours that affect risk of dental caries. This pilot study also appears to be among the first studies that captures information on dietary behaviours related to dental caries in a Canadian university campus population.

Overall, our pilot study found that dietary behaviours related to dental caries were relatively common in a Canadian university campus sample. For example, we found that consumption of sugary beverages was common. More specifically, we also found that consumption of other sugary drinks (e.g., iced tea, sports or energy drinks, sweetened iced coffee, iced cappuccinos, etc.) was more common than consumption of juice and pop (i.e., other sugary drinks: 53.8% of campus participants reported consumption a few times per week or more often; juice: 36.7% of campus participants reported consumption a few times per week or more often; pop: 20.3% of campus participants reported consumption a few times per week or more often). In addition, we also found that 41.3% of campus participants consumed a high risk sugary food item at least a few times per week or more often. Other studies have also found that dietary behaviours generally known to be associated with dental caries are also common in university student populations. For example, Kunitomo et al. [23] in a study focusing on oral health found that 24.2% of first year university students at a university in Japan consumed sugar-sweetened soft drinks frequently. In addition, Luebke and Driskell [24] found that ~64.8% of students at a university in the USA reported consuming 12 oz of pop 1-3 times/week or more often. They also found that among students who consumed pop, most reported a preference for regular pop. In addition, these authors also found that ~61.2% of participants reported eating sugary foods daily. These findings suggest that dietary behaviours associated with dental caries may be common in campus populations and further study in this area in Canada with a larger sample size is warranted to better understand this problem. In addition, our pilot study results also suggest that capturing information on a variety of sugary beverages other than just pop and juice should be a priority in this line of research.

One finding from our pilot study that was particularly noteworthy was that afternoon and evening snacks were the most commonly reported times of day for sugary food intake. Of note, Kunitomo et al. [23] also found that 23.8% of first year university students at a university in Japan reported frequent snacking and/or eating at night although they did not investigate the types of foods eaten during this time. The consumption of sugary foods in the evening is an important finding as previous research has found that consumption of free sugars close to bed time has been found to be associated with dental caries in children. For example, Goodwin et al. [25] found that in children aged 12–13 years, those who consumed ≥1 sugary snack before bed had a statistically significant higher mean decayed missing filled tooth (D_4-6_MFT) score compared to those who did not (1.5 vs. 0.7). In addition, Levine et al. [26] found in children that consumption of non-milk extrinsic sugars at bed time was positively associated with dental caries. The reason for this relationship is thought to be due to decreased saliva rates which cause a drop in plaque pH [25].

Daily or more frequent consumption of plain cow’s milk was low in both groups, and especially the campus group. Of note, 33.3% of campus participants reported consuming “other milks” at least a few times per week or more often. Had we considered only cow’s milk, data for those consuming non-dairy milk alternatives would not have been captured. Interestingly, soy milk was not widely reported to be consumed in our study. Although Canadian data are not available, sales of almond milk alone grew over 250% in the United States from 2011 through 2015 [27]. While these beverages may be consumed as an alternative to cow’s milk, nutrient profiles of individual products vary greatly with respect to components impacting dental health such as vitamins, minerals, fat and protein [28]. These products also differ in fortification status and added sugars. Previous research has also found non-dairy milk beverages exhibit some cariogenic properties. For example, Lee et al. [29] found that soy beverages and sugar-sweetened almond milk beverages exhibited cariogenic properties in vitro (e.g., *S. mutans* biofilm formation, low pH values). In addition, Huang et al. [30] also found that there was variation in the cariogenic properties of different non-dairy milk beverages with soy, almond, macadamia, cashew, and coconut being potentially more cariogenic and flax milk being potentially less cariogenic. Healthcare providers should be aware of these differences and should advise patients accordingly. Future research is still needed to better understand the relationship between non-dairy milk beverages and dental caries in clinical trials and epidemiological studies. In addition, we also found that sugary yogurt was more commonly consumed than non-sugary yogurt in the campus group; in both groups, intake of cheese was common. We did not attempt to determine at what times sugary dairy or milk alternatives were consumed versus non-sugary varieties of these items and cheese, as we did not separate these categories in our questionnaire. As dairy products may offer protective benefit, client education around consuming these items at snack times or at the end of meals, particularly where free sugar is also consumed, may be beneficial.

Despite the prevalence of dental caries in Canada and within this study’s sample (approximately four-fifths of each group had a history of caries and around two-fifths had caries diagnosed by a dental professional within the past 2 years), many participants indicated that they had never received advice from a healthcare professional regarding dietary risk factors for dental caries (more than half of the campus group, and almost half of the clinic group). This finding is not surprising, as offering nutrition counseling in dental practices is associated with substantial barriers, and few dental professionals refer patients to dietitians or nutritionists for nutrition counseling [31,32,33,34]. In addition, nutrition training in dental schools has been found to be limited and there have been many challenges reported with providing this type of education [35,36]. More inter-professional collaboration between dental professionals and dietitians, as well as education on nutrition and oral health in dietetic and dental training programs will undoubtedly help to decrease the burden of dental caries. Tools such as the one developed in this study could potentially help increase dialogue on this topic in various clinical settings with the aim of decreasing dental caries overall.

Overall, the questionnaire showed good test-retest reliability. Two negative kappa values were observed for responses to one nominal, select-all-that-apply type question. These type of questions received the lowest kappa scores in our study, and have also been found to perform worst and receive negative kappa values in other studies we reviewed [37,38].

This questionnaire appears to be one of the first of its kind—a practical assessment tool that healthcare providers can integrate into practice to encourage dietary counselling around specific, individualized caries risk factors. Further, it is easily modified for use in whole or in part, and designed for adults and children, supporting nutrition care for dental caries across the lifespan. It is a versatile tool which we hope will be further refined for integration into practice and research.

### Limitations

As neither sample were randomized, results cannot be generalized to other populations. Additionally, risk of voluntary bias response tends to be high in surveys as those with strong interests in nutrition or dental health may have responded. However, excluding nutrition and dentistry students, faculty and professionals and incentivizing participation may have helped to attenuate this concern. Due to the small size of the clinic sample, we were not able to perform tests of significance between participant groups, and therefore cannot make inferences regarding differences between groups.

Content validity was assessed via preliminary expert review; however, further work is required to assess the validity of this questionnaire against other dietary assessments methods. A next step would be validation against weighed food records, diet diaries, 24-h recalls, and/or food frequency questionnaires to determine the relative accuracy of this tool.

## 5. Conclusions

Dietary behaviours that may increase risk for dental caries were reported in both samples. Given the high prevalence of dental caries in this study, and low reporting of previous nutrition advice for the same, preventive education on dietary risk factors for dental caries may be warranted in university campus settings. In addition, our pilot study results suggest that further research on the relationship between dietary intakes and dental caries in Canadian university campus populations is needed. The questionnaire appears to show good initial reliability. Further work is also required to refine and validate this tool against commonly used dietary assessment methods to strengthen its further application.

## Figures and Tables

**Table 1:** Participant Demographic Characteristics.

	Campus *n* (%)	Clinic *n* (%)
Sex		
Female	60 (75.0)	7 (70.0)
Male	20 (25.0)	3 (30.0)
Age (years)		
6–13	0 (0.0)	3 (30.0)
18–34	65 (81.3)	2 (20.0)
35–54	14 (17.5)	2 (20.0)
55 and older	1 (1.3)	3 (30.0)
Position on Campus		
Undergraduate student	36 (45.0)	-
Graduate student	27 (33.8)	-
Staff/Faculty/Post-Doctoral Fellow	12 (15.0)	-
Other	4 (5.0)	-
Prefer not to answer	1 (1.3)	-
Newcomer to Canada within the past 5 years		
No	65 (81.3)	7 (77.8)
Yes	13 (16.3)	2 (22.2)
Prefer not to answer	2 (2.5)	0 (0.0)
Self-reported history of dental caries		
No	10 (12.8)	2 (22.2)
Yes	67 (85.9)	7 (77.8)
Prefer not to answer	1 (1.3)	0 (0.0)
Self-reported history of caries diagnosed by a dental professional within the past 2 years		
No	36 (53.7)	4 (57.1)
Yes	28 (41.8)	3 (42.9)
Do not know	3 (4.5)	0 (0.0)

Note: Numbers vary as not all participants submitted answers for each question.

**Table 2:** Sugar consumption behaviours among participants in the Campus and Clinic groups.

	Campus *n* (%)	Clinic *n* (%)
Usual daily intake pattern—sugary foods		
Breakfast	27 (33.8)	2 (20.0)
Morning snack	14 (17.5)	1 (10.0)
Lunch	10 (12.5)	1 (10.0)
Afternoon snack	32 (40.0)	7 (70.0)
Evening meal	21 (26.3)	1 (10.0)
Evening snack	41 (51.3)	3 (30.0)
Consumption of one or more high-risk items (dried fruit/fruit snacks, sugary candies, mints or lozenges)		
Rarely or never	9 (11.3)	1 (10.0)
A few times per month or less	38 (47.5)	5 (50.0)
A few times per week	25 (31.3)	3 (30.0)
Once per day	8 (10.0)	1 (10.0)
Addition of sugar to food and drinks		
No	45 (57.0)	4 (40.0)
Yes	34 (43.0)	6 (60.0)

Note: Numbers vary as not all participants submitted answers for each question.

**Table 3:** Consumption behaviours of food and beverage items that may be associated with increased or reduced dental caries risk among participants in the Campus and Clinic groups.

Frequency of Consumption	Sugary Non-Dairy Beverages						Dairy – Sugar Sweetened				Non Dairy Milk Beverages				Dairy – No Sugar Added					
	Juice		Pop/Soda		Other Sugary Drinks		Flavoured Cow’s Milk		Yogurt		Soy Milk		Other Milks (Almond, Rice, Hemp, Coconut, etc.)		Cow’s Milk		Cheese		Yogurt	
	Campus *n* (%)	Clinic *n* (%)	Campus *n* (%)	Clinic *n* (%)	Campus *n* (%)	Clinic *n* (%)	Campus *n* (%)	Clinic *n* (%)	Campus *n* (%)	Clinic *n* (%)	Campus *n* (%)	Clinic *n* (%)	Campus *n* (%)	Clinic *n* (%)	Campus *n* (%)	Clinic *n* (%)	Campus *n* (%)	Clinic *n* (%)	Campus *n* (%)	Clinic *n* (%)
Rarely or never	50 (63.3)	5 (50.0)	63 (79.7)	7 (70.0)	37 (46.3)	5 (50.0)	70 (92.1)	8 (80.0)	42 (55.3)	3 (33.3)	72 (93.5)	4 (100.0)	52 (66.7)	9 (100.0)	37 (48.1)	3 (30.0)	16 (20.5)	1 (10.0)	47 (61.8)	4 (40.0)
A few times per week	25 (31.6)	4 (40.0)	14 (17.7)	3 (30.0)	35 (43.8)	4 (40.0)	3 (3.9)	2 (20.0)	26 (34.2)	6 (66.7)	5 (6.5)	0 (0.0)	14 (17.9)	0 (0.0)	22 (28.6)	2 (20.0)	38 (48.7)	9 (90.0)	15 (19.7)	5 (50.0)
Once per day	4 (5.1)	0 (0.0)	2 (2.5)	0 (0.0)	6 (7.5)	1 (10.0)	2 (2.6)	0 (0.0)	8 (10.5)	0 (0.0)	0 (0.0)	0(0.0)	9 (11.5)	0 (0.0)	12 (15.6)	3 (30.0)	20 (25.6)	0 (0.0)	13 (17.1)	1 (10.0)
More than once per day	0 (0.0)	1 (10.0)	0 (0.0)	0 (0.0)	2 (2.5)	0 (0.0)	1 (1.3)	0 (0.0)	0 (0.0)	0 (0.0)	0 (0.0)	0 (0.0)	3 (3.8)	0 (0.0)	6 (7.8)	2 (20.0)	4 (5.1)	0 (0.0)	1 (1.3)	0 (0.0)

Note: Numbers vary as not all participants submitted answers for each question.

**Table 4:** Nutrition Questionnaire Test-Retest Reliability Results.

Item	Question Text	Question Format	Weighted Kappa	Cohen’s Kappa	Absolute Agreement (%)
1.	Each day, when do you usually have drinks, including smoothies? Do not include water, unsweetened black coffee or tea, or other zero-calorie drinks.	Check all that apply			
	Breakfast			0.654	83.0
	Morning Snack			0.314	83.0
	Lunch			0.393	77.4
	Afternoon Snack			0.339	71.7
	Evening Meal			0.350	73.6
	Evening Snack			0.508	77.4
2.	Each day, when do you usually have food?	Check all that apply			
	Breakfast			0.694	88.7
	Morning Snack			0.685	84.9
	Lunch			-0.047	90.6
	Afternoon Snack			0.508	75.5
	Evening Meal			-0.026	94.3
	Evening Snack			0.427	71.7
3.	When do you usually eat sugary or sweet foods such as cereals, cookies, cakes, baked goods, chocolate bars, fruit in syrup, or other sugary foods?	Check all that apply			
	Breakfast			0.488	77.4
	Morning Snack			0.554	86.7
	Lunch			0.291	86.8
	Afternoon Snack			0.279	64.2
	Evening Meal			0.063	73.6
	Evening Snack			0.469	73.6
	Rarely or Never			0.466	84.9
4.	How often do you usually drink each of the following?	Categorical frequency			
	Juice (100% juice or other fruit drinks)		0.735		84.6
	Pop/Soda (regular or reduced-sugar but not diet or zero-calorie)		0.714		90.4
	Other sugary drinks (iced tea, sports or energy drinks, sweetened iced coffee, iced cappuccinos, etc.)		0.597		76.9
5.	Do you add sugar to food or drinks? Include: white or brown sugar, molasses, honey, agave, other natural or processed syrup, and other natural sugars such as cane, coconut or palm sugar. Do not include calorie-free sweeteners such as aspartame, stevia or sucralose.	Yes/No		0.674	84.9
6.	Do you chew sugary gum, or eat any of the following: dried fruit, chewy fruit snacks, sugary candies (gummy or hard), breath mints or throat lozenges?	Categorical Frequency	0.560		66.0
7.	How many servings of vegetables and fruits do you eat each day? (Select the closest answer) One serving of vegetables is ½ cup of fresh, frozen or cooked vegetables, ½ cup of cooked greens or 1 cup of raw leafy greens One serving of fruit is one whole piece of fruit or ½ a cup of chopped or frozen fruit (not fruit juice or juice canned in syrup				
	Vegetable servings	Categorical frequency	0.514		50.9
	Fruit servings	Categorical frequency	0.627		59.1
8.	How often do you usually drink the following types of milk? (Please specify a number if indicated). Include milk that you have with cereal.	Categorical frequency			
	Plain cow’s milk		0.707		75.0
	Flavoured cow’s milk		0.788		95.9
	Soy milk (any type)		0.503		90.2
	Other milks		0.834		90.6
9.	If you drink soy milk or other milk (such as almond, rice, hemp, coconut, cashew, etc.), has sugar been added?	Multiple choice		0.711	83.0
10.	If you drink soy milk or other milk (such as almond, rice, hemp, coconut, cashew, etc.), has it been fortified with vitamins and minerals?	Multiple choice		0.645	78.8
11.	When do you usually have milk (any of the types mentioned above)?	Check all that apply			
	Breakfast			0.764	90.6
	Morning Snack			0.387	86.8
	Lunch			0.731	96.2
	Afternoon Snack			0.497	90.6
	Evening Meal			0.590	86.8
	Evening Snack			0.705	90.6
	Rarely or Never			0.767	94.3
12.	How often do you usually eat cheese or yogurt (made from cow’s or goat’s milk, not soy or other ingredients)?	Categorical frequency			
	Cheese		0.673		71.1
	Plain or no added sugar yogurt		0.672		77.6
	Sugar sweetened yogurt		0.759		86.3
13.	When do you usually have cheese or yogurt (only the types listed above)?	Check all that apply			
	Breakfast			0.722	86.8
	Morning Snack			0.466	84.9
	Lunch			0.660	83.0
	Afternoon Snack			0.357	75.5
	Evening Meal			0.409	71.7
	Evening Snack			0.515	81.1
	Rarely or Never			0.560	92.5

## Supplementary Materials

The following are available online at https://www.mdpi.com/1660-4601/17/5/1793/s1, Figure S1: Final Questionnaire.
