# Supplementary material for: Development of a Nutrition Questionnaire for Dental Caries Risk Factors
Source: Int J Environ Res Public Health. 2020 Mar 10;17(5):1793. doi: 10.3390/ijerph17051793 (PMC7084613; doi:10.3390/ijerph17051793)
Supplement: Supplementary file 1 [file ijerph-17-01793-s001.pdf]

Figure S1: Final Questionnaire

Note: #10 omitted in clinic sample; #15 omitted in campus sample.

Please select the answer that best describes your habits on **most days** over the **past 3 months**. Parents/caregivers – if completing this form for a child, answer from the child's perspective.

1. Each day, when do you usually have **drinks**, including smoothies? (Check all that apply)

Do not include water, unsweetened black coffee or tea, or other zero-calorie drinks.

□ Breakfast	□ Lunch	□ Evening meal
□ Morning snack	□ Afternoon snack	□ Evening snack
□ Other times (please specify):_______________________		

2. Each day, when do you usually have **food**? (Check all that apply)

□ Breakfast	□ Lunch	□ Evening meal
□ Morning snack	□ Afternoon snack	□ Evening snack
□ Other times (please specify):_______________________		

3. When do you usually eat **sugary or sweet foods** such as cereals, cookies, cakes, baked goods, chocolate bars, fruit in syrup, or other sugary foods? (Check all that apply)

□ Breakfast	□ Lunch	□ Evening meal
□ Morning snack	□ Afternoon snack	□ Evening snack
□ Other times (please specify):__________________________		□ Rarely or never

4. How often do you usually **drink** each of the following? (Please specify a number if indicated)

**Juice (100% juice or other fruit drinks)**	□ Rarely or never	□ A few times per week: __________	□ Once per day	□ More than once per day: __________
**Pop/Soda (regular or reduced-sugar but not diet or zero-calorie)**	□ Rarely or never	□ A few times per week: __________	□ Once per day	□ More than once per day: __________

**Other sugary drinks (iced tea, sports or energy drinks, sweetened iced coffee, iced cappuccinos, etc.)**	□ Rarely or never	□ A few times per week: __________	□ Once per day	□ More than once per day: _________

5. Do you add sugar to **food or drinks**?

Include: white or brown sugar, molasses, honey, agave, other natural or processed syrup, and other natural sugars such as cane, coconut or palm sugar.
Do not include calorie-free sweeteners such as aspartame, stevia or sucralose.

□ No
□ Yes – how many teaspoons (or packets) do you usually add per day? _________

6. Do you **chew** sugary gum, or **eat** any of the following: dried fruit, chewy fruit snacks, sugary candies (gummy or hard), breath mints or throat lozenges?

□ No
□ Yes – how often do you have **at least one** of the above items?
□ A few times per month or less □ A few times per week □ Once per day □ More than once per day

7. How many **servings** of vegetables and fruits do you eat each day? (Select the closest answer)

One serving of vegetables is ½ cup of fresh, frozen or cooked vegetables, ½ cup of cooked greens or 1 cup of raw **leafy** greens.
One serving of fruit is one whole piece of fruit or ½ a cup of chopped or frozen fruit (**not fruit juice or juice canned in syrup.**

**Vegetables**	□ 0	□ 1	□ 2	□ 3	□ 4	□ 5 or more
**Fruit**	□ 0	□ 1	□ 2	□ 3	□ 4	□ 5 or more

8. How often do you usually drink the following types of **milk**? (Please specify a number if indicated)

Include milk that you have with cereal.

**Plain cow's milk (white, any fat content)**	□ Rarely or never	□ A few times per week: __________	□ Once per day	□ More than once per day: _________
**Flavored cow's milk (chocolate, vanilla, strawberry, etc.)**	□ Rarely or never	□ A few times per week: __________	□ Once per day	□ More than once per day: _________
**Soy milk (any type)**	□ Rarely or never	□ A few times per week: __________	□ Once per day	□ More than once per day: _________
**Other milks (almond, rice, hemp, coconut, cashew, etc.)**	□ Rarely or never	□ A few times per week: __________	□ Once per day	□ More than once per day: _________

9. If you drink soy milk or other milk (such as almond, rice, hemp, coconut, cashew, etc.), has **sugar been added**?

□ No □ Yes □ Don't know □ Not applicable

10. If you drink soy milk or other milk (such as almond, rice, hemp, coconut, cashew, etc.), has it been fortified with **vitamins and minerals**?

□ No □ Yes □ Don't know □ Not applicable

11. When do you usually have **milk** (any of the types mentioned above)? (Check all that apply)

□ Breakfast □ Lunch □ Evening meal
□ Morning snack □ Afternoon snack □ Evening snack
□ Other times (please specify):_________________________ □ Rarely or never

12. How often do you usually eat **cheese or yogurt** (made from cow's or goat's milk, **not** soy or other ingredients)? (Please specify a number if indicated)

**Cheese (any type)**	□ Rarely or never	□ A few times per week: __________	□ Once per day	□ More than once per day: _________
**Yogurt (regular or Greek):**				

**Plain, no added sugar or artificially unsweetened**	□ Rarely or never	□ A few times per week: ___________	□ Once per day	□ More than once per day: __________
**Sugar sweetened**	□ Rarely or never	□ A few times per week: ___________	□ Once per day	□ More than once per day: __________

. 13. When do you usually have cheese or yogurt (**only** the types listed above)? (Check all that apply)

□ Breakfast □ Lunch □ Evening meal
□ Morning snack □ Afternoon snack □ Evening snack
□ Other times (please specify):___________________________ □ Rarely or never

. 14. Do you have any questions or concerns about food and dental cavities? □ No □ Yes - please list: ____
____
____

. 15. For those answering on behalf of a child **only** – does the child use either of the following?

Bottle: □ No □ Yes – what drinks is this used for? (Please list)__________________________
Sippy cup: □ No □ Yes – what drinks is this used for? (Please list)__________________________

Not applicable □
